# Temperature Effects Removal from Non-Stationary Bridge–Vehicle Interaction Signals for ML Damage Detection

**DOI:** 10.3390/s23115187

**Published:** 2023-05-30

**Authors:** Sardorbek Niyozov, Marco Domaneschi, Joan R. Casas, Rick M. Delgadillo

**Affiliations:** 1 Department of Structural, Geotechnical and Building Engineering, Politecnico di Torino, 10129 Turin, Italy; sardorbek.niyozov@studenti.polito.it; 2 Department of Civil and Environmental Engineering, Universitat Politècnica de Catalunya, 08034 Barcelona, Spain; joan.ramon.casas@upc.edu; 3 Department of Civil Engineering, Universidad de Ingenieria y Tecnologia—UTEC, Jr. Medrano Silva 165, Barranco, Lima 15063, Peru

**Keywords:** bridge damage detection, non-stationary, PCA, K-means, operational-environmental variability

## Abstract

Bridges are vital components of transport infrastructures, and therefore, it is of utmost importance that they operate safely and reliably. This paper proposes and tests a methodology for detecting and localizing damage in bridges under both traffic and environmental variability considering non-stationary vehicle-bridge interaction. In detail, the current study presents an approach to temperature removal in the case of forced vibrations in the bridge using principal component analysis, with detection and localization of damage using an unsupervised machine learning algorithm. Due to the difficulty in obtaining real data on undamaged and later damaged bridges that are simultaneously influenced by traffic and temperature changes, the proposed method is validated using a numerical bridge benchmark. The vertical acceleration response is derived from a time-history analysis with a moving load under different ambient temperatures. The results show how machine learning algorithms applied to bridge damage detection appear to be a promising technique to efficiently solve the problem’s complexity when both operational and environmental variability are included in the recorded data. However, the example application still shows some limitations, such as the use of a numerical bridge and not a real bridge due to the lack of vibration data under health and damage conditions, and with varying temperatures; the simple modeling of the vehicle as a moving load; and the crossing of only one vehicle present in the bridge. This will be considered in future studies.

## 1. Introduction

Aging, material degradation, deterioration, fatigue, and corrosion are some of the reasons structures lose their performance. They can sometimes cause structures to fail unexpectedly. The research in this area [1] has increased as major disasters have occurred worldwide [2], resulting in the death of a considerable number of people and large economic consequences. In this regard, structural health monitoring (SHM) systems are becoming an important tool to prevent structural failures with direct and indirect losses.

A useful classification of SHM was proposed by Rytter [3], who categorized four levels of damage assessment depending on the characteristics of damage that a particular SHM system can achieve.

- Level 1: Indication of damage existence (Detection);
- Level 2: Information of damage position (Localization);
- Level 3: Damage intensity (Assessment);
- Level 4: Prognosis (Remaining life prediction).

With the advancement of machine learning (ML) algorithms, a new level that corresponds to the type of damage can be introduced above [4]. This new level stands between Levels 2 and 3.

Vibration data has typically been used to detect bridge deterioration based on changes in modal parameters brought on by the presence of damage. It is rather easy to obtain dynamic parameters as modal ones and damping properties using traditional time-frequency signal processing techniques such as fast Fourier transform (FFT) and short-time Fourier transform (STFT) [5,6]. Such signal processing techniques, however, are predicated on the idea that the signal is stationary and linear, which may be argued in general to be valid for the duration of the short time window, but is not valid over the entire timespan of the observation. In addition, FFT presents a few intrinsic limitations that can hinder the effectiveness of the system identification and the following damage detection [7]. For instance:

(i) The structural health state may be lost during the data reduction procedure, which is specifically retrieved by FFT [8].
(ii) FFT cannot identify the time dependence of the dynamic parameters, and when using signals from naturally excited structures, it is unable to capture the evolutionary traits that can be observed in these signals [9].

The higher frequencies are poorly excited and closely spaced, and they typically represent damages that are local events [10,11]. As a result of the linearity and stationarity assumptions, damage detection methods using FFT show relevant limitations. The signals derived from vehicles crossing a bridge, however, do not exhibit these properties because the frequency spectrum of the response shifts along the crossing due to the vehicle-bridge interaction and because the signal’s amplitude is modified by the vehicle’s position on the bridge in relation to the location where the response is recorded.

It becomes clear that the recorded vibration’s frequency and amplitude are time dependent. As a result, techniques for modal parameter extraction (such as FFT) that rely on the linearity and stationarity of the process are inapplicable for obtaining trustworthy and precise damage indicators.

Wavelet-based methods, addressed in the literature and applied to bridges, allow for valuable insights with respect to modal identification. However, the developed techniques are aimed at the identification of general time-varying systems, while it is known that other critical conditions such as the vehicle-bridge interaction contain closely spaced spectral components due to the vehicle–bridge dynamic coupling, which instead requires alternative approaches [12].

Two major limitations of SHM during continuous monitoring are also present. First, by applying increasingly sophisticated algorithms to monitoring data, the number of features that are sensitive to degradation are estimated rather than being measured directly. Natural frequencies, for instance, are determined through measurements of vibrational reactions, such as accelerations, which are subject to estimation mistakes. In addition to changes brought on by structural degradation, the features discovered through monitoring are also sensitive to operational (traffic load) and environmental (temperature, humidity, and wind speed) conditions. As a result, in order to properly apply SHM, it is necessary to consider the accuracy of the predicted features as well as environmental and operational variability.

In conclusion, since environmental and traffic effects always have an impact on dynamic response in real bridges in use (causing non-linearity and non-stationarity in the recorded signals), there are two main challenges when using damage detection techniques in active bridges: how to eliminate ambient effects and extract precise damage features that are practical for damage detection, location, and quantification from short non-linear and non-stationary vibration produced when vehicles cross the bridge. This paper will demonstrate how relevant damage features derived from vibration records and machine learning techniques can deliver a relevant answer to the query.

## 2. Damage Detection Method

It is generally recognized that a number of external elements, including the environment and operational circumstances (traffic), do have effects on the efficiency of SHM systems when adopted for bridges. Several SHM works from the literature are focused on the use of machine and statistical learning techniques to address this issue. For instance, when the temperature is constant, Santos et al. [13] suggested an online unsupervised detection technique for early damage identification. Additionally, Soo et al. [14] proposed a procedure for distinguishing environmental effects from damage in close to real-time using principal component analysis, although without taking the operational loading into account. They do not, however, offer a complete solution for bridges that are subject to both traffic loads and temperature fluctuations. Tenelema et al. [15] showed that principal component analysis (PCA) is efficient for differentiating environmental effects from selected data when traffic data are used. They used instantaneous phase differences as damage-sensitive features on a steel bridge and demonstrated that the first principal component (PC) is purely related to temperature effects. Even though the temperature effect was clearly shown, the bridge FEM model did not accurately represent a real bridge structure. Moreover, the damage detection was based on the variance changes in the second PC of PCA, which cannot guarantee certainty in real cases because of excessive noise.

Delgadillo and Casas [16] demonstrated on a real steel truss bridge that instantaneous frequencies (IF) are reliable damage-sensitive features by applying the Hilbert-Huang transform and mode decomposition, performing unsupervised cluster-based machine learning (K-means) on symbolic data. As a result, K-means proved to be efficient to detect and localize the damage, but temperature effects were not considered. Therefore, the K-means algorithm is a promising tool to be tested in further developments that include temperature effect removal.

More recent contributions from the literature can also be reported. Wang et al. [17] proposed a hybrid method for damage detection under environmental changes. Specifically, they used a PCA and Gaussian mixture method to cluster the data into damaged and undamaged states.

Huang et al. [18] presented a “train-based performance warning method” for bridge main girders using long-term data, considering temperature, wind, and traffic load. However, their objective is more focused on removing the traffic effects from the analyzed vibration record rather than on using the capability of vibration due to traffic for damage detection.

Meixedo et al. [19,20] considered together different environmental and operational effects on bridges, i.e., temperature, wind, and train load. In particular, they used the portion of the vibration response when the load is on the bridge for damage detection purposes.

Zhang et al. [21] proposed a steady-state data baseline model for bridges by eliminating the non-stationary effects of the temperature by employing PCA directional projection. Strain data at the different points of the bridge were used.

Wang et al. (2022) [22] discussed the displacement model error-based performance warning to detect structural anomalies using PCA to eliminate traffic loads, assuming the first PC only to be related to the traffic loads.

This paper’s goal is to provide an SHM methodology for a bridge under operational (traffic loading) and environmental (temperature) effects. Therefore, in this study, both conditions are considered together, while the contributions by Tenelema et al. and Delgadillo and Casas [15,16] do not deal completely with both effects and do not provide an appropriate threshold to define the presence of damage. With respect to Wang et al. [17], who argued that the main components carry information about the state of the structure, this paper reconstructs the data by removing first the PC, related to temperature effects, and then identifies damage by using an advanced machine learning approach. Conversely, Huang et al. [18] adopted PCA to remove wind and traffic effects, while temperature was eliminated using a canonically correlated method. Zhang et al. [21] applied a machine learning approach to the second PC for damage detection without considering other PCs and environmental effects, while the present work employs K-means for detecting and localizing damage. Moreover, considering the contribution by Wang et al. (2022) [22], where long-span bridges and static data only are considered, this paper focuses on standard and frequent bridges with short and medium spans and uses dynamic response.

Finally, with respect to Meixedo et al. [19,20], where the vibration due to trains was used to obtain autoregressive (AR) and autoregressive exogenous input (ARX) models as feature extractors and used for damage detection only by fusing the data from different sensors, the main novelty of the present work is the vibration decomposition to deal with the intrinsic non-stationarity of the dynamic response induced by highway traffic to define instantaneous damage indicators in the frequency domain, with the main advantage that they may be used for both detection and localization of damage based on the results of different sensors.

**Figure 1:**
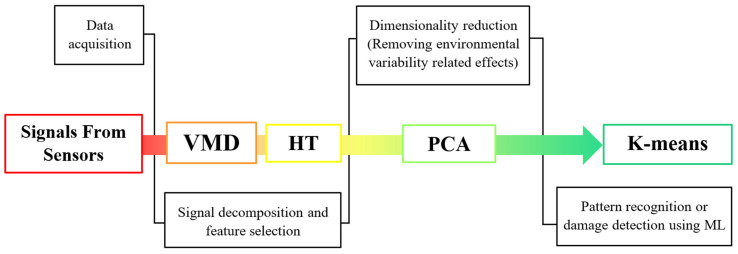
Flow chart of the proposed SHM methodology.

This study treats recorded non-stationary signals of the vehicle-bridge interaction in a comprehensive way, proposing PCA analysis for the removal of temperature effects and instantaneous frequencies as damage features. Furthermore, it includes the use of a damage index, based on a machine learning approach, as a clear threshold for differentiating damaged and undamaged scenarios. Figure 1 shows a flow chart of the proposed methodology, which is summarized as follows:

1. Acceleration records under traffic loads and at different ambient temperatures are collected from sensors.
2. Variational mode decomposition (VMD) and Hilbert transform (HT) are applied to recorded data in order to choose a damage-sensitive feature for the following step. This step aims to deconstruct the non-stationary signal into intrinsic mode functions (IMFs). In this study, instantaneous frequency is considered as the relevant damage feature.
3. Principal component analysis is performed to remove the environmental effects (temperature).
4. Symbolic data analysis is performed, and a cluster-based moving window K-means algorithm is applied to selected scenarios for damage detection and localization.
5. In the following subsections, the above-mentioned numerical tools are described in detail.

### 2.1. Variational Mode Decomposition (VMD)

Since Dragomiretskiy and Zosso’s 2014 [23] proposal, variational mode decomposition (VMD) has been extensively utilized in a variety of applications. By concurrently extracting modes, they devised a variational mode decomposition model that is completely non-recursive. It was developed to overcome empirical mode decomposition’s (EMD) limitations of sensitivity to noise and sampling.

VMD is frequently used in structural engineering for modal identification by means of the recorded dynamic response. For instance, Bagheri et al. [24] used a variety of case studies (numeric, experimental, and on site) to show the effectiveness of the VMD method. A field study of a pedestrian bridge looked at the structural response to ambient vibrations input, while a laboratory investigation focused on the vibratory response of a multi-story structure. In addition, the shear frame’s modal characteristics were calculated using an analytical method and then compared to the modal parameters computed by laboratory tests. As a result, compared to conventional signal decomposition techniques such as EMD, the VMD-based system identification was shown to be robust against noise signals and sampling frequency.

The limitation of this method is that the number of modes *K* must be set in advance [25]. If the selected mode number is not accurate, the VMD will cause the loss of important modes. In order to determine the number of intrinsic mode functions (IMFs) different methods have been proposed such as the correlation coefficient method [26], the normalized mutual information method [27], and, the most common, the center frequency observation method [28].

VMD’s objective is to discreetly divide an input signal with real values, *f*, into quasi-orthogonal band sub-signals with band limits, $u_{k}$ (modes). Each mode is condensed around the central pulsation $\omega_{k}$, and the bandwidth is calculated using the shifted signal’s $H^{1}$ Gaussian smoothness [28]. The constrained variational problem form of the VMD is as follows:

(1) $$\underset{\left\{u_{k}\right\},\left\{\omega_{k}\right\}}{min}\left\{\;\overset{K}{\underset{k=1}{\sum }}\|\partial_{t}\left[\left(\delta \left(t\right)+\frac{j}{\pi t}\right)*u_{k}\left(t\right)\right]e^{-j\omega_{k}t}{\|}_{2}^{2}\right\}$$

(2) $$s.\;t.\;\overset{K}{\underset{k=1}{\sum }}u_{k}=f$$

where $u_{k}$ and $\omega_{k}$ are the *k*th intrinsic mode function, and it is the center frequency, respectively; $\partial_{t}$ is the time derivative; δ(t) is Dirac’s delta function; and *j* is an imaginary number.

(3) $$ℒ\left(\left\{u_{k}\right\},\left\{\omega_{k}\right\},\lambda \right)=\alpha \underset{k}{\sum }\|\partial_{t}\left[\left(\delta \left(t\right)+\frac{j}{\pi t}\right)*u_{k}\left(t\right)\right]e^{-j\omega_{k}t}{\|}_{2}^{2}+\|f\left(t\right)-\underset{k}{\sum }u_{k}\left(t\right){\|}_{2}^{2}+\langle \lambda \left(t\right),f\left(t\right)-\underset{k}{\sum }u_{k}\left(t\right)\rangle$$

where the data fidelity constraint’s balancing parameter, α, is also known as the penalty factor.

### 2.2. Hilbert Transform (HT)

The HT is a linear operator that transforms a real signal x(t) into another real signal, indicated by H[x(t)]. It is defined as the convolution of x(t) with the function 1/(πt):

(4) $$H\left[x_{k}\left(t\right)\right]=\frac{1}{\pi }P.V\int_{-\infty }^{+\infty }\frac{x_{k}\left(\tau \right)}{t-\tau }d\tau$$

P.V is Cauchy’s principal value of the integral, and $x_{k}\left(t\right)$ corresponds to the *k*th IMF component obtained from the mode decomposition technique. For simplicity purposes, the notation $x_{k}\left(t\right)$ is used instead of $IMF_{k}\left(t\right)$ in all equations below. The HT allows defining the complex analytic signal $z_{k}\left(t\right)$, from which instantaneous amplitude and phase can be computed. An analytic signal represents rotation in the complex plane with the rotation radius $a_{k}\left(t\right)$ and the instantaneous function $\theta_{k}\left(t\right)$ [25]. This implies that the analytic signal becomes:

(5) $$z_{k}\left(t\right)=x_{k}\left(t\right)+i\;H\left[x_{k}\left(t\right)\right]=a_{k}\left(t\right)e^{i\theta_{k}\left(t\right)}$$

where $H\left[x_{k}\left(t\right)\right]$ represents the Hilbert transform of $IMF_{k}\left(t\right)$, $a_{k}\left(t\right)$ is the instantaneous amplitude, and $\theta_{k}\left(t\right)$ is the instantaneous phase function.

(6) $$a_{k}\left(t\right)=\sqrt{{\left\{x_{k}\left(t\right)\right\}}^{2}+{\left\{H\left[x_{k}\left(t\right)\right]\right\}}^{2}}$$

(7) $$\theta_{k}\left(t\right)=arctan\left(\frac{H\left[x_{k}\left(t\right)\right]}{x_{k}\left(t\right)}\right)$$

The instantaneous amplitude $a_{k}\left(t\right)$ describes the envelope of the denoised $IMF_{k}\left(t\right)$, while $\theta_{k}\left(t\right)$ describes the number of rotations.

The concept of the frequency and phase carry significant importance when applying IMFs [29]. If the IMFs can be considered local, then the instantaneous angular frequency $\omega_{k}\left(t\right)$ can be defined as:

(8) $$\omega_{k}\left(t\right)=\frac{d\theta_{k}\left(t\right)}{dt}=2\pi f_{k}\left(t\right)$$

Cohen [30] stated that each IMF is a mono-component signal with a monotonically increasing phase and a positive instantaneous frequency. Therefore, each selected IMF can be defined as:

(9) $$x_{k}\left(t\right)=Re(z_{k}\left(t\right))=Re\left(a_{k}\left(t\right)e^{i\theta_{k}\left(t\right)}\right)=a_{k}\left(t\right)\cos\left[\theta_{k}\left(t\right)\right]$$

The sum of the instantaneous phases for the chosen IMFs can be used to determine the overall instantaneous phase:

(10) $$\theta \left(t\right)=\underset{k}{\sum }\theta_{k}\left(t\right)=\underset{k}{\sum }arctan\left(\frac{H\left[x_{k}\left(t\right)\right]}{x_{k}\left(t\right)}\right)$$

where $x_{k}\left(t\right)$ are physically meaningful (i.e., closer to the natural frequencies) IMFs selected for spectral analysis, and θ(t) is the total number of rotations in the complex plane, in radians (rad), of a significant part of the original measured signal with xk(t).

### 2.3. Clustering Algorithm

The study of grouping or clustering things based on measurable or perceived intrinsic features or similarities is known as cluster analysis. Data clustering (unsupervised learning) differs from classification or discriminant analysis since it lacks category information (supervised learning). Clustering techniques do not require defining reference/training data, in contrast to supervised algorithms. By looking for the smallest and most distinct group of clusters, they may “understand” the structure of a data set [31].

The primary objective of clustering algorithms is to identify a structure or a pattern in the data. It is an effort to increase the dissimilarity between objects assigned to various clusters while simultaneously minimizing the distinction between data objects mathematically associated with the same cluster [31]. There is no univocal belief on the whole definition of clustering, although a traditional one is provided below [32]:

1. Instances, in the same cluster, must be similar as much as possible.
2. Instances, in the different clusters, must be different as much as possible.
3. Measurement for similarity and dissimilarity must be clear and have practical meaning.

K-means is taken into account in this work. It is a partition-based clustering approach. This class of algorithms’ fundamental principle is to treat each data point’s center as the center of its associated cluster [32].

Let $P_{K}=\left\{C_{K},\;\ldots ,\;C_{K}\right\}$ be partitioned into *K* clusters. Then, the overall within-cluster $W\left(P_{K}\right)$ dissimilarity can be defined as [31]

(11) $$W\left(P_{K}\right)=\frac{1}{2}\overset{K}{\underset{k=1}{\sum }}\underset{c\left(i\right)=k}{\sum }\underset{c\left(j\right)=k}{\sum }d_{ij}$$

where c(i) is a many-to-one allocation function that assigns object i to cluster k, with respect to a dissimilarity measure, $d_{ij}$, defined between each pair of data objects, i and j.

The overall dissimilarity of a data set, OD, is given below.

(12) $$OD=\frac{1}{2}\overset{N}{\underset{i=1}{\sum }}\overset{N}{\underset{j=1}{\sum }}d_{ij}$$

where N is the total number of objects. The between-cluster dissimilarity is obtained by subtracting the previous two equations, $B\left(P_{K}\right)=OD-W\left(P_{K}\right).$

The goal of the K-means is to minimize the overall within-cluster dissimilarity, $W\left(P_{K}\right)$, of a given partition, $P_{K}$, by the iterative optimization scheme. The K-means requires that the number of *K < N* clusters be initially defined [31] with a randomly defined set of *K* cluster prototypes from the same type of data. This step is called initialization. Following the initialization, each iteration starts by assigning objects to clusters by allocating rule, c(i). The second step of the K-means algorithms is to find the best prototypes that represent clusters defined before. It is called the representation step. K-means represents the clusters by finding their centroids. The process of allocation and representation is continued until an objective function that is dependent on the compactness and separation of the clusters reaches its global minimum value [31].

The K-means uses squared within-cluster dissimilarity measured across the *K* clusters as an objective function [32]. They are usually based on distance metrics. Euclidean distance is the most widely utilized one (square root of the sum-of-squares). However, it causes significant false detection occurrences and increases computing complexity when SHM is applied. The alternate Gowda-Diday distance measure is employed to go through these issues [31].

### 2.4. Damage Identification

The last step in the process is the definition of a quantitative value (a threshold value or detection index) that clearly defines the limit between the undamaged and damaged situations and minimizes the number of false positives.

The K-means algorithm demands the initial definition of the number of K≤N of clusters, as well as a randomly defined set of cluster prototypes, which are objects of the same type as those being clustered. The process of choosing which set of prototypes best depicts the clusters specified during the allocation phase is known as the representation step of each K-means iteration. There is no way to predict in advance if this number will match the number of distinct structural conditions identified on site. Clear conclusions cannot be formed without identifying which of the partitions best capture the data’s structure. The global silhouette index (*SIL*) is employed in this study to obtain the number of clusters since it performed better in prior investigations when its formulation was thoroughly discussed [13,33]. The *i*th observation is given a set number of clusters *K*, with the following value, to create the silhouette statistic [16]:

(13) $$s\left(x_{i}\right)=\frac{b\left(x_{i}\right)-a\left(x_{i}\right)}{\max\left\{a\left(x_{i}\right),\;b\left(x_{i}\right)\right\}}\in \left[-1,1\right]$$

where $a\left(x_{i}\right)$ is the average distance between the ith object of cluster *C* and the remaining *j* objects, and $b\left(x_{i}\right)$ the distance to the center of the closest neighboring cluster. Below are shown, accordingly, the average silhouette widths for all samples and the silhouette index of clusters, where $\left(1\leq M_{k}\leq N\right)$, *N* is the set of objects, *K* is the partition for clustering, and the silhouette coefficient ranges from 0 to 1.

(14) $$s\left(x\right)=\frac{1}{M_{k}}\overset{M_{k}}{\underset{i=1}{\sum }}s\left(x_{i}\right)$$

(15) $$SIL=\frac{1}{K}\overset{K}{\underset{k=1}{\sum }}s\left(x\right)$$

For the purposes of the current study, it is significant to note that the partition that, among the *K* evaluated, has the highest *SIL* value is the one that best fits the examined data set and should therefore be taken into consideration for SHM.

Cluster analysis can automatically distinguish between structural situations without making any assumptions about the site’s past structural condition. However, user input is required to determine whether the output is compact or mixed over time.

It creates several difficulties to implement the method in real-time applications. To overcome this issue, Santos et al. [31] proposed a robust strategy for damage detection using a cluster-based algorithm with moving time windows. It relies on the average difference between clusters rather than relying on the allocation of data objects to cluster over time by defining the following damage index *DC* [31].

(16) $$DC=\frac{1}{K\left(K-1\right)}\overset{K}{\underset{k=1}{\sum }}\overset{K}{\underset{\begin{array}{c} c=1\\ c\neq 1 \end{array}}{\sum }}d_{ck}$$

*c* and *k* are two of the *K* clusters, *K* is the number of clusters from the partition with the highest *SIL*, and *d_ck_* is the Gowda-Diday dissimilarity calculated between their centroids. The K-means clusters are similar and will produce low values of *DC* if there is no damage and the structural behavior is stable. Therefore, the K-means algorithm will produce distinct and independent clusters and high values of the *DC* if damage is detected. Santos et al. [31] further contend that *DC* is particularly sensitive to early damage and capable of large data fusion because the information gathered from numerous sensors is referred to as a single-value index.

Ideally, TRUE/FALSE binary information should be provided by the detection strategy. *L* is the time duration of each data sample, S is the number of samples per window, and *SL* is the fixed length in which the time windows are specified. Santos et al. [31] propose the statistical testing of the *DC* values acquired inside each time window’s length because the value of *DC* is not informative by itself (it does not provide TRUE/FALSE information). The statistical testing of the *DC* values results in the definition of the confidence boundary (*CB*), which should be exceeded only in the case the structural system exhibits changes.

The *CB* is defined at each time window, by statistically testing *DCs* distribution, under the assumption that only the random effects influence the residual errors obtained from unchained structures. Generally, normal statistical distribution is used in SHM works related to damage detection. To describe small samples taken from a Gaussian population, the *t*-student distribution is more suited because there are just a few *DC* values in each time window [31].

The confidence boundary at each time window is obtained as follows:

(17) $$CB=E\left[DC\right]+t_{s-1,\;\frac{1}{2}+\frac{\beta }{2}}\times E\left[DC-E\left[DC\right]\right]/\sqrt{S}$$

where $t_{s-1,\;\frac{1}{2}+\frac{\beta }{2}}$ is the $\frac{1}{2}+\frac{\beta }{2}$ percentile of a *t*-student distribution with *S* − 1 degrees of freedom, and *β* is the confidence level taken at 99.9%.

E[DC] and E[DC−E[DC]] are expected value and variability estimates of the *DC* sample within the analyzed time window, respectively.

Finally, Santos et al. [31] suggested an original detection index, *DI*, based on *DC* and *CB* values. It is defined to have the following properties: (i) it is dimensionless, (ii) it has an unsupervised and window-wise character by using only data pertaining to a single time window, and (iii) it has positive values that indicate damage detection (“TRUE”) and negative or null values that stand for unchanged structural response (“FALSE”). As defined by the following, *DI* (detection index) is defined as the ratio between the median *DC* (damage index) value observed within a time interval and the largest *DC* deviation observed above the *CB* (confidence boundary).

(18) $$DI=\frac{max_{i}\left(DC_{i}-CB\right)}{med_{i}\left(DC_{i}\right)};\;\;i=1,\;\ldots ,\;S$$

## 3. Case Study

### 3.1. Description of the Concrete Bridge Model

The proposed methodology is applied to a numerical model of a two-span continuous beam bridge’s superstructure component under changing operating and environmental conditions. The model was developed starting from an original benchmark steel bridge presented by Tatsis and Chatzi [34] via open-source Python scripts, which were made available through GitHub (Table 1), with the objective of representing better actual bridge structures.

**Figure 2:**
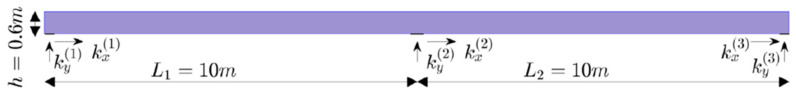
Geometry of the two-span bridge in the longitudinal direction with elastic boundaries.

The bridge superstructure (Figure 2) consists of a two-span bridge with equal length (*L*_1_ and *L*_2_ = 10 m). The cross-section of the beam is rectangular with a constant width of 10 m and a height of 0.6 m.

The bridge is assumed to be made from reinforced concrete with *E* = 35 GPa. It is a linear elastic material with density *ρ* = 2400 kg/m^3^ at ambient temperature *T* = 20 °C and Poisson’s ratio *ν* = 0.2. Table 1 summarizes the geometry and the mechanical properties.

The bridge is supported by nine elastomeric rubber bearings that are modeled as three evenly spaced elastic supports at each abutment and intermediate pier and put on the bottommost edge of the bridge superstructure.

Table 2 summarizes the global horizontal stiffness, *k_x_*, and vertical stiffness, *k_y_*, adopted for the three spring supports that consider the stiffness of the corresponding elastomeric bearings designed according to the loads acting on the bridge (self-weight, permanent load, traffic load, temperature, etc.).

### 3.2. Damage Scenarios and Sensors

**Figure 3:**
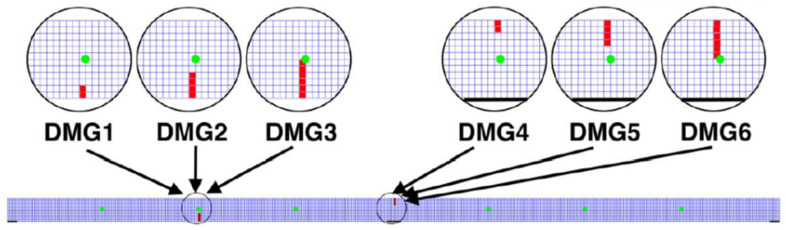
Sensors (in green) and damage locations (in red) in bridge structure.

In order to provide information about the nodal variables in both the *x-* and *y-* directions, six sensing points, or “sensors,” are thought of (i.e., displacements, velocities, accelerations, strains, etc.). Table 3 describes where these sensors are located. Figure 3 displays the six chosen sensors as green points. Furthermore, six damage scenarios (DMG1 to DMG6) are modeled in the bridge. Damages are introduced by assigning lower stiffness characteristics to selected series of elements. In the following, they are termed “damage-induced mesh elements” (red elements in Figure 3).

The first damage region is found in the center of the left span (DMG1, DMG2, DMG3), starting from the bottommost edge of the beam cross-section, while the second damage region is found in the intermediate support section (DMG4, DMG5, DMG6), starting from the uppermost edge of the beam. Moreover, the number of damage-induced mesh elements also varies, as shown in Figure 3. For instance, DMG1 and DMG4 cover an area of two damaged elements, DMG2 and DMG5 a zone of four damaged elements, while DMG2 and DMG6 a zone of six damaged elements. As a result, the damaged elements are 0.05 m wide and range in height from 0.1 to 0.3 m. Table 4 provides a summary of the descriptions of these six damage situations.

In addition to the damage scenarios, the model allows controlling the severity of the damage by setting the reduction of Young’s modulus.

Stiffness reduction is noted by D, followed by the amount of reduction. For example, D50% means 50% reduction in Young’s modulus at a given element and damage scenario.

### 3.3. Loading

**Figure 4:**
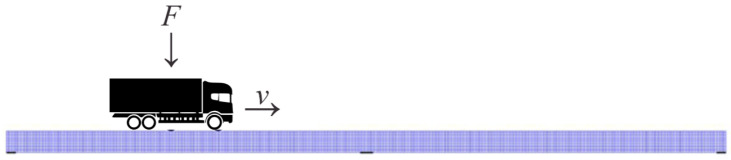
Loading in the form of a moving vertical force *F* with a constant speed *v*.

To perform a time-history analysis simulating traffic flow, a deterministic moving load is used. Data processing methods such as the Hilbert Huang transform can detect these singularities in the signal caused by vibrations created by the moving load and is the appropriate tool to analyze the non-stationary accelerations produced by the crossing load along the bridge [29,30]. The passage of a vehicle is modeled as a moving load F with constant speed v in this case study, as shown in Figure 4. The weight of a standard truck of about 30 tons is considered.

In summary, the following parameters are considered for the time-history analysis:

− The first, second, and third bending modes are selected;
− The Rayleigh damping coefficients are *α* = 0.1654 and *β* = 5.4333 × 10^−6^;
− The vehicle velocity is 10 m/s;
− A sampling frequency of *f_s_* = 400 Hz in the virtual sensors, a time step in the time domain analysis of Δ𝑡 = 0.001 s, a final time step *T_f_* = 2 s (time to cross the bridge), and a total number of time samples of 800 are considered.

#### Simulation of Temperature

To check the ability of the machine learning methodology proposed to overcome the problems related to the temperature effects in the recorded signals, different responses are obtained under different ambient temperatures. This is introduced in the numerical model by taking into account the relationship between temperature and Young’s modulus of concrete. The work by Jiao et al. [35] was assumed, where a linear relationship between the modulus of elasticity and temperature was given:

(19) $$E_{c}=-0.125T+29.13$$

(20) $$R^{2}=0.9852\;$$

For the present study $E_{c}=35\;\mathrm{GPa}$ is taken at a reference temperature of 20 °C, and the relationship is modified accordingly.

(21) $$E_{c}=-0.125T+37.5$$

### 3.4. Results and Discussion

As stated earlier, the main objective of this paper is to propose and test the machine-learning-based methodology for damage detection in bridges that will be able to distinguish the real damage from the environmental changes and using the forced vibrations due to the traffic crossing. To simulate those conditions, it was decided to model a passing of the truck at two different temperatures and in both damaged and undamaged states.

Therefore, the following extreme scenarios for time-history response due to traffic action were preliminarily chosen in the present work:

1. UND, T = −15 °C, D0%;
2. UND, T = 20 °C, D0%;
3. DMG3, T = 20 °C, D90%;
4. DMG3, T = −15 °C, D90%.

In all cases, the damage is introduced at the mid-span of the left span.

**Figure 5:**
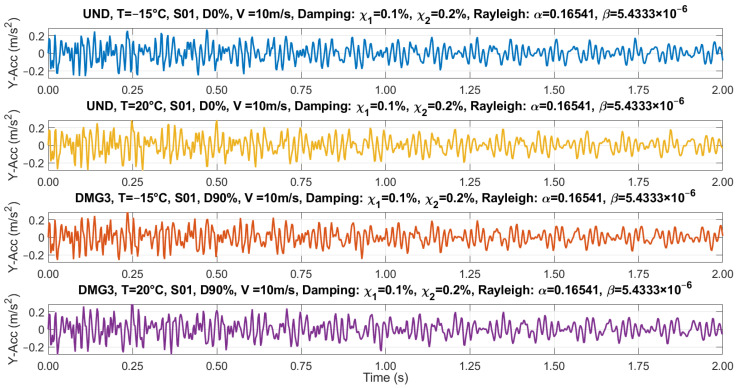
Recorded Y-accelerations at S01.

Figure 5 shows the obtained accelerations at Sensor S01 for the four considered scenarios.

#### 3.4.1. VMD

VMD is a signal decomposition technique with its own experimental parameters. Since the IMFs reveal the frequency information contained in the original signal, the peaks in the Fourier spectrum of this signal give a rough indication of the number of IMFs to be extracted from a particular signal decomposition technique. Therefore, the single-sided amplitude spectrum of the decomposed IMFs is also discussed.

There are six parameters that must be set to apply VMD $\varepsilon_{a},\;\varepsilon_{r},\;O,\;\alpha ,\;\tau \;\mathrm{and}\;K$. $\varepsilon_{a}\;\mathrm{and}\;\varepsilon_{r}$ are absolute and relative tolerance, respectively. In this case study, the absolute tolerance is more restrictive than the relative tolerance, as in the implementation of MATLAB. Therefore, $\varepsilon_{a}=0.1$ and $\varepsilon_{r}=10^{-5}$. A high number of iterations O=100,000 is set to stop the VMD only when relative tolerance is met.

**Figure 6:**
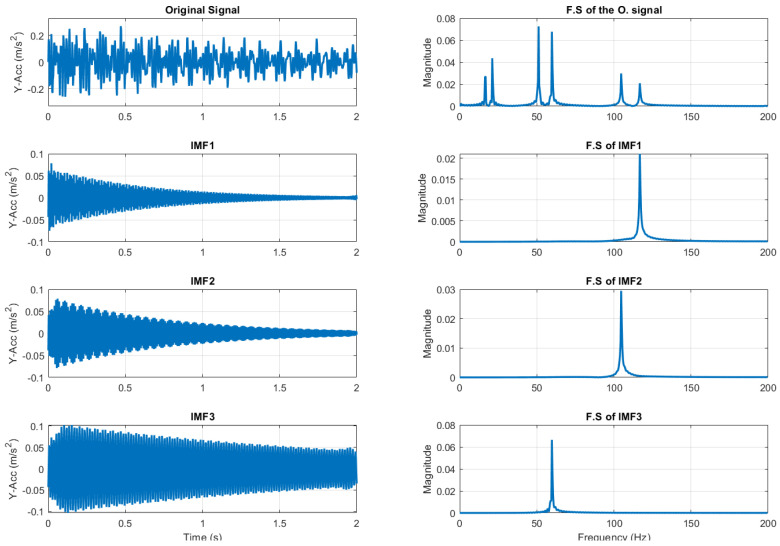

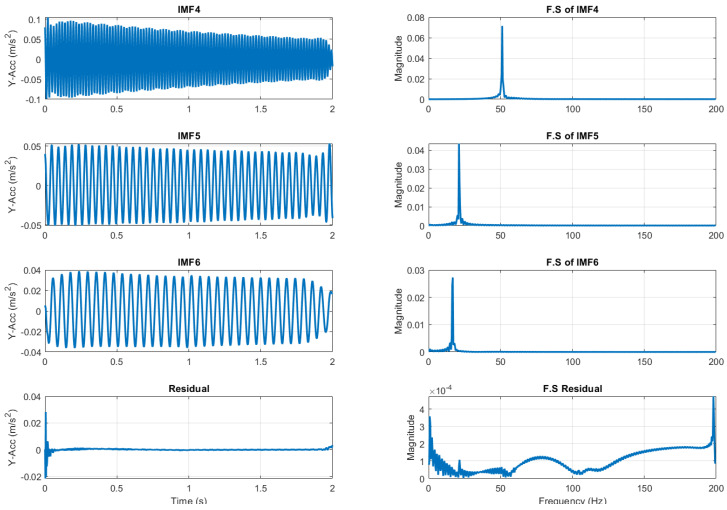
Y-acceleration signal of undamaged state from S01 at T = 20 °C and its VMD.

The efficiency of the VMD method has been demonstrated. The algorithm always seeks to reconstruct the time series effectively with minimum error. The VMD method ensures no mixture of modes, as well as the orthogonality of the transformation. Although this section refers to the signal decomposition for the undamaged state at T = 20 °C in Sensor S-01 (Figure 6), the same procedure is performed for all sensors and compared to the damage scenarios. This study revealed that the signal decomposition is similar for every damage scenario using the same VMD parameters as in Sensor S01: *ε_a_* = 0.1, *ε_r_* = 10^−5^, 𝜏 = 0.1, *ε_r_* = 10^−5^ and O = 100,000, with the exception of the number of modes *K*, which varies from one sensor to another, as shown in Table 5.

**Figure 7:**
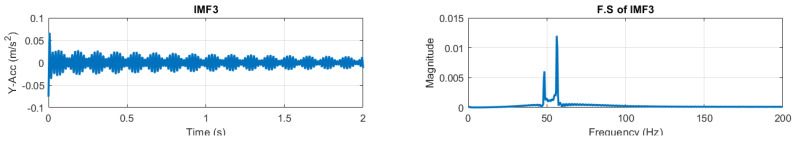
IMF 3 of Y-acceleration signal of undamaged state from S02 at T = 20 °C.

*K* is found to be 6 for Sensors S01, S03, S04, and S06, which are located at ¼ from the spring supports, whereas *K* is established as 5 for Sensors S02 and S05, which are placed in the middle of the left span and right span, respectively. In these sensor signal amplitudes, the second bending mode is far less than in the rest of the sensors, which results in a mix of the asymmetric and symmetric modes, as shown in IMF3 in Figure 7.

#### 3.4.2. Hilbert Transform

The Hilbert transform is applied to study the characteristics of different time-varying parameters obtained from scenarios and to obtain damage-sensitive features. In particular, the goal is to examine each IMF and obtain instantaneous frequency as a function of time. It can be achieved by applying the Hilbert transform (HT) to each IMF extracted from the application of the VMD method.

**Figure 8:**
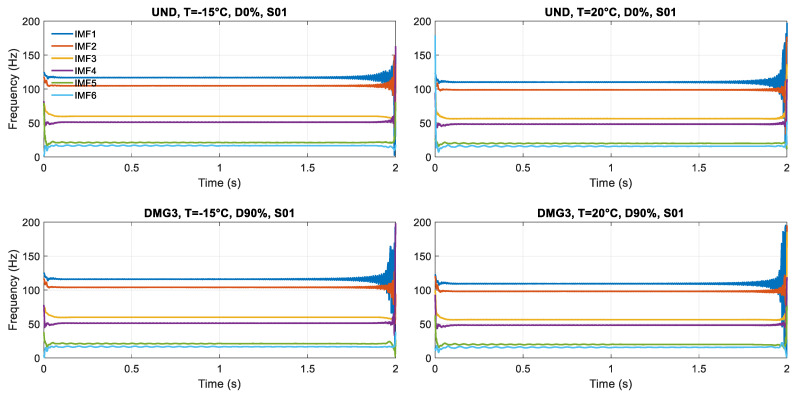
Instantaneous frequency for each scenario in Sensor S-01.

**Figure 9:**
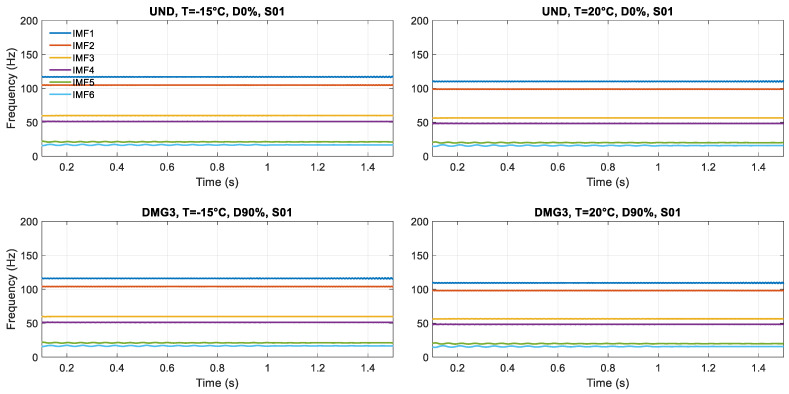
Concatenated instantaneous frequencies for each scenario in Sensor S-01.

The instantaneous frequencies $f_{k}\left(t\right)$ are obtained by applying the Hilbert transform to the *k* physically meaningful IMFs, e.g., Figure 8 for Sensor S-01. It can be noticed how boundary conditions affect the response: when the load is at the extremities, over the abutments (time instant of 0 s and 2 s), larger oscillations are generated in the model because of the load concentration and the elastic properties (no dissipative effects) of the numerical model. Therefore, it was decided to concatenate the analysis on the instantaneous frequencies in the interval of 0.1–1.5 s as the appropriate time signal for the analysis (Figure 9).

#### 3.4.3. Application of PCA (Eliminating Environmental Effects)

When PCA [14,36,37,38] is applied to the data set, the first principal component (PC1) represents the factor that creates the greatest variance within the data set, the second principal component (PC2) represents the factor that creates the second greatest variance affecting the data, and so on.

According to Soo et al. [37], the data set needs to come from two extreme and opposing temperature situations in order to accurately depict the impacts of temperature fluctuations in PC1. As a result, if the data set is standardized, the extreme cases will be displayed on the opposing sides of the PC1 graph, indicating negative and positive values of the variance. Therefore, additional minor elements affecting the data set, such as structural damages, will be represented by the other primary components.

**Figure 10:**
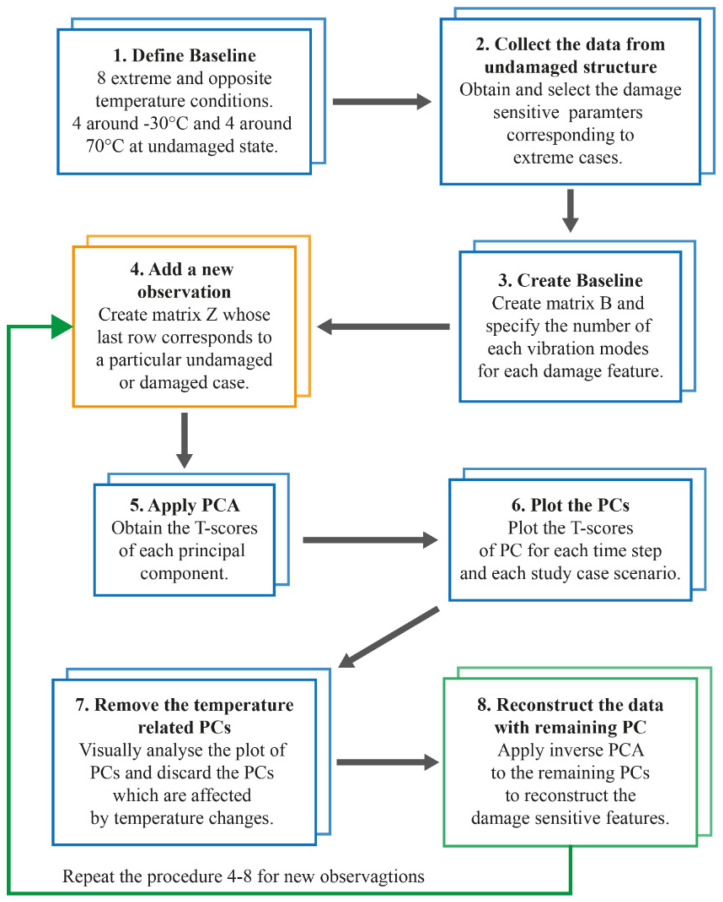
Flow chart of the PCA-based filtering method.

As shown in the flowchart in Figure 10, the PCA methodology to filter the temperature effects impacting the vibration parameters may be broken down into eight primary steps.

The basic data set for typical PCA is a 2-D matrix, $X_{n,m}$, consisting of *n* observations (e.g., temperature variability) and *m* measured variables (e.g., modes). The correlation between these variables is examined using the standard PCA method. However, because the observed variables (e.g., instantaneous frequencies) are continuous in time in this case study, a third dimension, *k*, is added, resulting in $X_{n,m,k}.$

**Figure 11:**
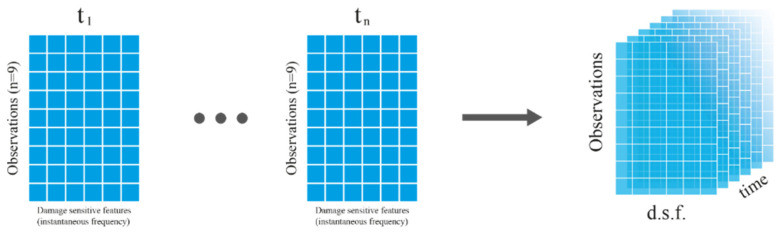
Unfolding of PCA matrix for a sensor.

In this case study, correlations between a particular measured variable (instantaneous frequency) obtained for each IMF *m* and several temperature observations, *n*, are analyzed. Therefore, each time sample and each sensor are treated independently. As an illustration, Figure 11 shows the unfolding in time samples of the data set corresponding to a sensor resulting in a 3-D data matrix, $X_{n,m,k}$.

**Figure 12:**
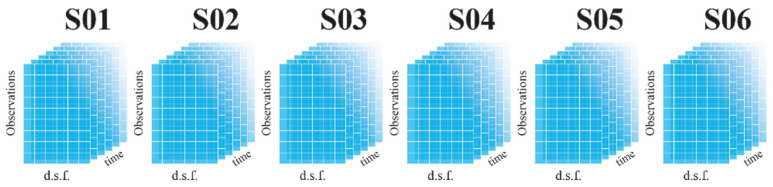
Complete data matrix for PCA.

Moreover, a fourth dimension, *l*, must be added to the data matrix when various sensors are examined. As a result, a four-dimensional data matrix $X_{n,m,k,l}$ with a high number of connected variables is created, as illustrated in Figure 12.

On the one hand, the number of observations is *n* = 9 corresponding to the 8 extreme temperatures (case observations) to create the baseline plus one new observation. The number of modes, m, represents the number of IMFs in which the original signal was decomposed. As seen in the previous section, the number of IMFs *m* varies from one sensor to another: *m* = 6 for Sensors S01, S03, S04, and S06, and *m* = 5 for Sensors S02 and S05. As a reminder, the instantaneous modal parameters were obtained for the interval of 0.1–1.5 s and a sampling frequency of 400 Hz was selected to capture the first three bending modes of vibration, hence dimension *k* = 560. The vertical acceleration response obtained for any temperature condition was decomposed by means of the VMD-based method with the following parameters: $\alpha =1000\;\varepsilon_{a}=0.1$, $\varepsilon_{r}=10^{-5}$, 𝜏 = 0.1, $\varepsilon_{r}=10^{-5}$ and *O* = 100,000.

**Figure 13:**
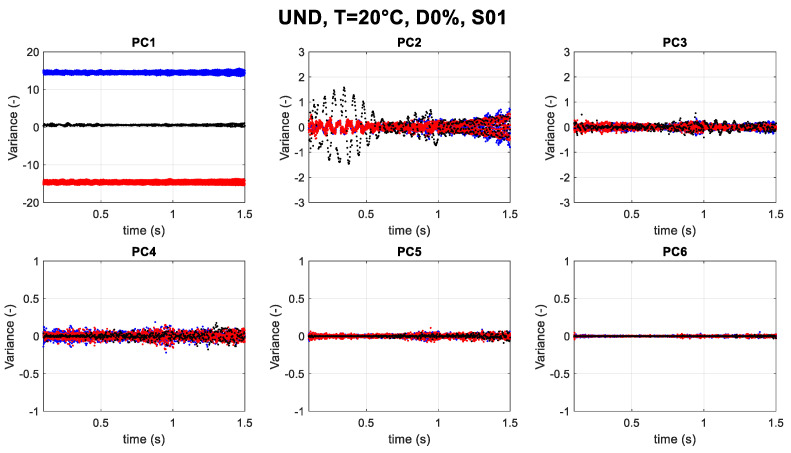
PCA of UND T = 20 °C, D0%, S01 (black dots); blue dots: extreme negative temperatures (−30 °C, −29 °C, −28 °C, −27 °C); red dots: extreme positive temperatures (67 °C, 68 °C, 69 °C, 70 °C).

**Figure 14:**
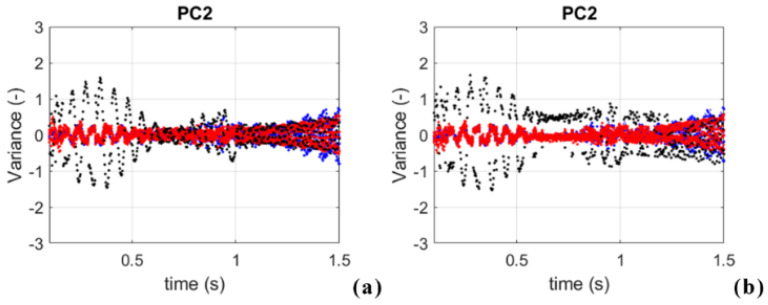
Comparison of PC2: (**a**) UND T = 20 °C, D0%, S01 (black dots); blue dots: extreme negative temperatures ( −30 °C, −29 °C, −28 °C, −27 °C); red dots: extreme positive temperatures (67 °C, 68 °C, 69 °C, 70 °C) (**b**) DMG3, T = 20 °C, D90%, S01 (black dots); blue dots: extreme negative temperatures (−30 °C, −29 °C, −28 °C, −27 °C); red dots: extreme positive temperatures (67 °C, 68 °C, 69 °C, 70 °C).

In the following, the graphical results for the undamaged cases and damaged cases corresponding to scenarios at 20 °C temperature are presented (black dots) in Figure 13 and Figure 14 (for PC2 only). These represent a representative sample of the results over different scenarios. Moreover, to create the baseline, four extreme cases at low temperatures of around −30 °C (blue dots) and four extreme cases at high temperatures of around 70 °C (red dots) are considered.

The instantaneous frequency $x_{k}\left(t\right)$ of each IMF was determined. Figure 13 illustrates the six components for the undamaged scenario cases of 20 °C for Sensor S01. On the vertical axis, the variance of the principal components (dimensionless) is reported. Furthermore, it can be noted that the magnitude of the observations in PC1 is much larger than that in PC2 for all considered scenarios.

As mentioned, PC1 related to the data shows the highest variance related to the temperature. As scenarios under consideration are at −15 °C and 20 °C, they both fall between extreme cases, both in damaged and undamaged conditions, thus making it clear that PC1 is definitively temperature related.

Moreover, it is important to note that PC2 can reveal some changes in the structure response as well. If two cases of damaged and undamaged conditions at T = 20 °C are compared (Figure 14), the difference in PC2 can be observed.

As a next step, the damage-sensitive features (instantaneous frequencies) are reconstructed using the five last principal components (from PC2 to PC6). It is worth noting that the reconstructed data should be rescaled to have a physical meaning.

**Figure 15:**
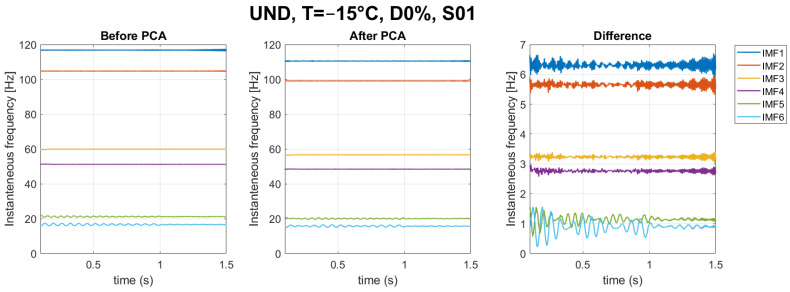
Instantaneous Frequencies before and after the application of PCA dimensionality reduction.

In Figure 15 below, the IF from a representative scenario (undamaged: −15 °C) at S01 is shown before and after the application of PCA dimensionality reduction. It is worth noting that IF at 20 °C had a lower change in magnitude between, after, and before the application of PCA with respect to −15 °C.

**Figure 16:**
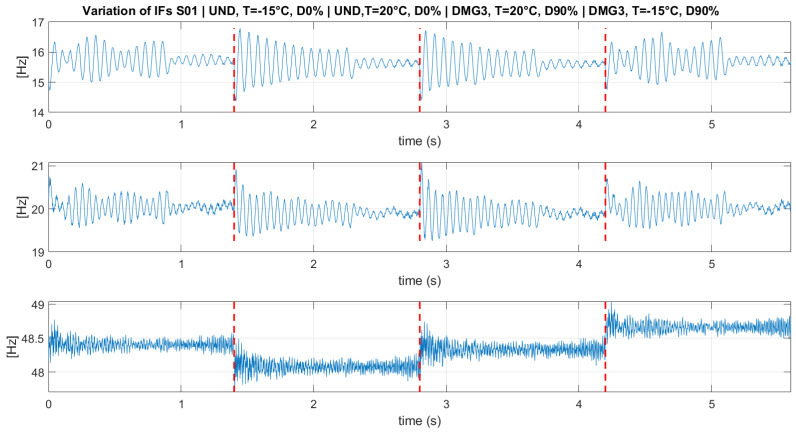

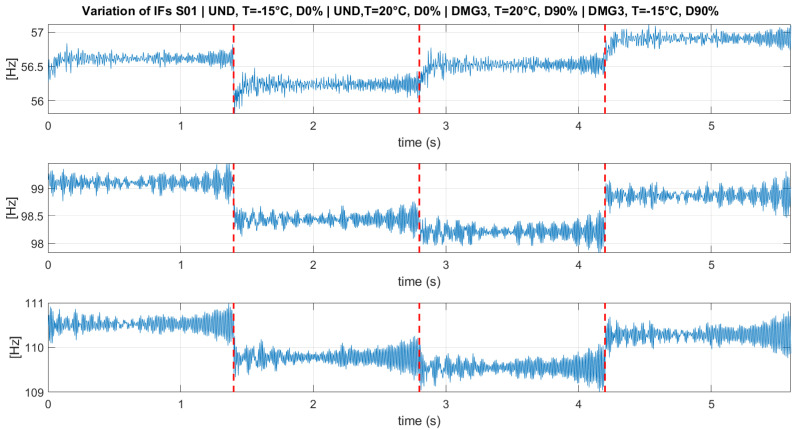
Variation of the instantaneous frequencies of different scenarios related to S01.

Figure 16 shows the variation of the instantaneous frequencies at each damage scenario from S01. As can be observed, although PCA dimensionality reduction was applied at high frequencies (~48 Hz, ~56.5 Hz, ~98.7Hz, ~110 Hz), the difference between damage and temperature scenarios in their value is visibly clear. The application of cluster analysis to such data will not be appropriate, as the goal of the methodology is to distinguish between damaged and undamaged states when the influence of the temperature is not easily observed. Moreover, signals from S02 and S05 were disregarded as well, as the mode mixing problem due to the VMD created data mixing after the PCA analysis, making instantaneous frequencies unreliable.

In conclusion, only the first instantaneous frequencies (~15.6 Hz) from S01, S03, S04, and S06 were considered for the clustering algorithm.

#### 3.4.4. K-Means

**Figure 17:**
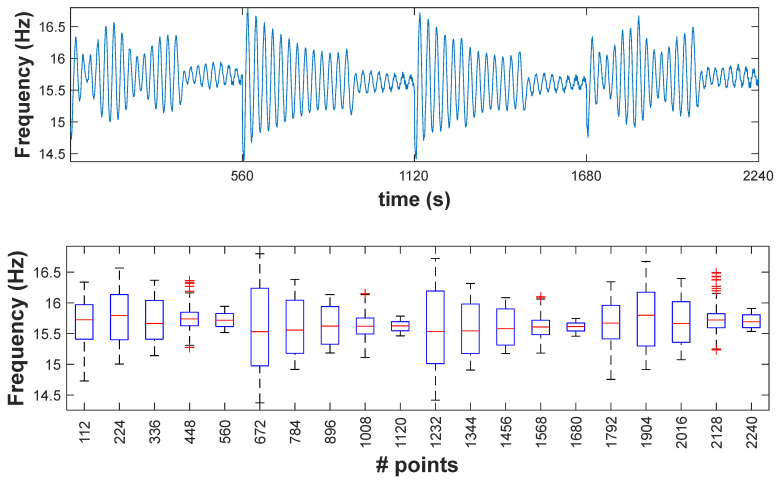
First instantaneous frequency of all scenarios for S01 and corresponding box plot.

Before the application of the K-means algorithm, the data must be reduced to more generic types and less voluminous information in contrast with classical data used in SHM. The extracted damage-sensitive features, in this case the instantaneous frequency of the four scenarios with a total time of 5.6 s, are converted into symbolic data by using the interquartile interval. The resulting total number of points was 2240. A symbolic data length of L = 112 points was considered, and a respective boxplot for each sensor was constructed (Figure 17).

Moreover, in the implementation of the K-means algorithm in MATLAB, the suitable method *“Start”* to determine the initial clusters centroid positions (or seeds) should be set among the options (Table 6).

For this study, the “sample” case was found as a consistent and appropriate method for the initial cluster centroid positions.

**Figure 18:**
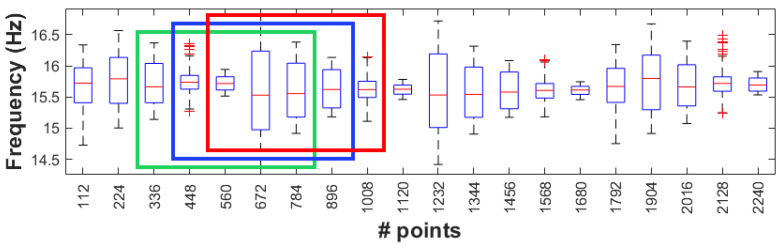
A sequence of mobile windows.

**Figure 19:**
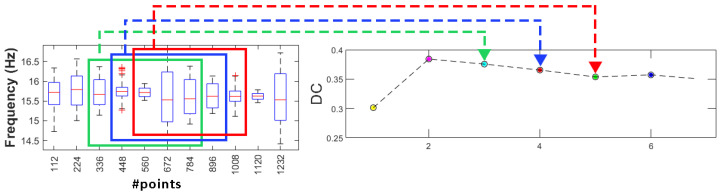
DC values obtained for each time window.

As explained, the detection method is based on the value of the *DC*, which is the difference between clusters. To calculate *DC*, the clusters of moving windows must be defined. In this work, the size of windows was selected as S = 5 symbolic data, each comprising L = 112 points of the damage-sensitive feature. Figure 18 shows a sequence of mobile windows, while Figure 19 shows the DC values obtained for each time window.

The total number of symbolic data equals 20, and the number of mobile windows and DC values is 16.

It should be noted that if the time window increases, then higher sensitivity to damage detection is obtained, but the time between damage occurrence and damage detection increases. Moreover, if the samples within a time window decrease, it is possible to detect the damage earlier, but the probability of false detections increases (there is less sensitivity to damage detection). Therefore, it is necessary to find a balance between the two objectives: detect damage as soon as possible but obtain high confidence in the detection.

After the *DC* values are obtained, they must be statistically tested by the confidence boundary test. *CB* is defined for each time window containing five values of *DC*.

Finally, using *DC* and *CB* values the original detection index, *DI* is calculated. When the *DI* value is negative or zero, there is no damage in the structure, and when it is positive, it indicates the presence of damage in the structure.

**Figure 20:**
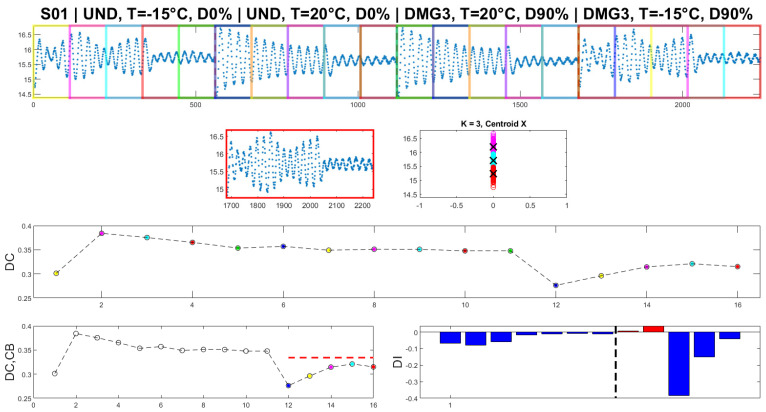
K-means clustering on the S01 first IF from different scenarios (Dashed line on the DI diagram: damage introduction).

Figure 20 shows the whole procedure for the IF (~15.6 Hz) from S01 for the damage scenarios. It can be seen that the clustering algorithm exactly detects damage (positive value of *DI*) when it crosses the scenarios between UND T = 20 °C D0% and DMG3 T = 20 °C D90% (marked with the black dotted vertical line). However, temperature change either in the undamaged or damaged scenarios does not create a positive *DI* value, therefore, not warning on the existence of damage (false detection).

**Figure 21:**
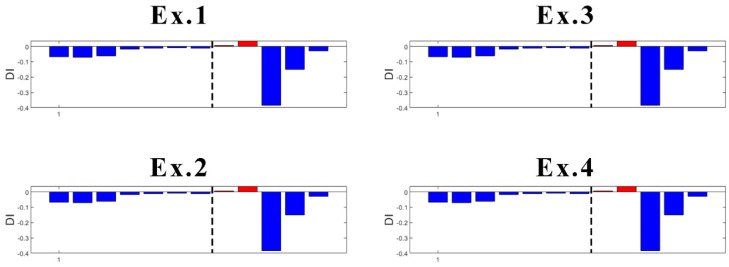
Experiments with DI for S01 (dashed line: damage introduction with 90% stiffness reduction).

One of the disadvantages of the K-means algorithm is its dependence on initial values. As the initial cluster centroids are assigned randomly, they may create different outputs each time the algorithm runs. However, in this case, this disadvantage was found useful to localize the damage. Specifically, an innovative aspect highlighted by this research is the following: K-means gives consistent detection of damage when using the data coming from sensor S01, the closest to DM3. In fact, as presented in Figure 21, four trials (experiments) of the algorithm gives always detection of damage when it appears, without false detections.

**Figure 22:**
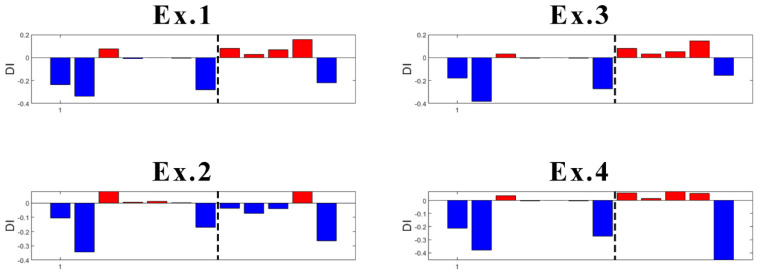
Experiments with DI for S03 (dashed line: damage introduction with 90% stiffness reduction).

In the case of sensor S03 (Figure 22), which is the second sensor closest to the damage, K-means provides early false detection in all cases. However, true damage detection is always obtained.

**Figure 23:**
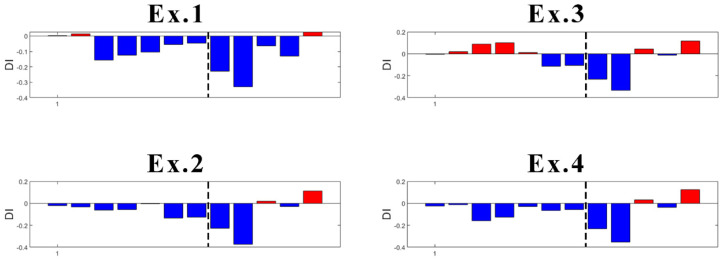
Experiments with DI for S04 (dashed line: damage introduction with 90% stiffness reduction).

**Figure 24:**
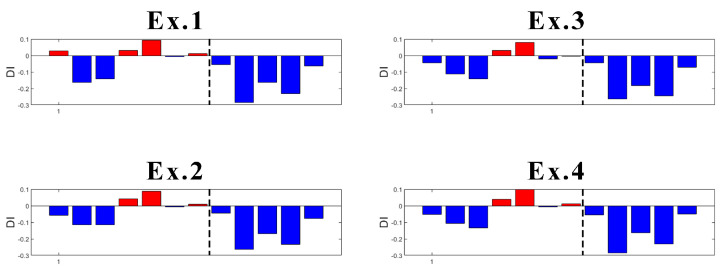
Experiments with DI for S06 (dashed line- damage introduction with 90% stiffness reduction).

What concerns the results of Sensors S04 and S06 located more far away from the damage location is that they both provided inconsistent results. With high randomness in their output, both early false detection and no detection of true damage can be seen in their results (Figure 23 and Figure 24).

Focusing on the results of DI (Figure 21 and Figure 22), it can be said that the damage is located somewhere between Sensors S01 and S03, which is correct as the damage was introduced at the mid-span of the left span. The results are not conclusive when looking at sensors far away from where the damage is present. Thus, as a further contribution to the present study, the proposed methodology is suitable not only for damage detection, but also for damage localization.

**Figure 25:**
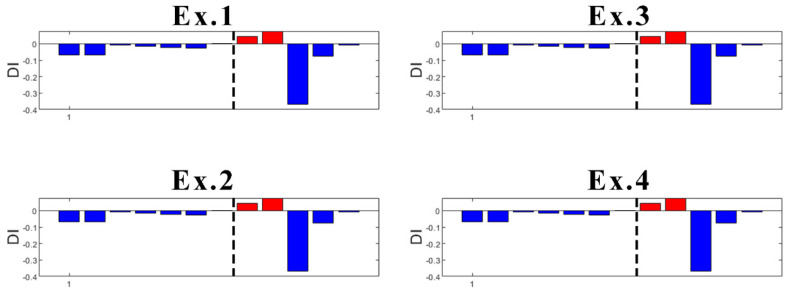
Experiments with DI for S01 (dashed line: damage introduction with 30% stiffness reduction).

Finally, the methodology was preliminarily tested out for the case with a smaller severity of damage (30% of reduction of E), considering Sensor S01 only and the same damage position already simulated for the previous analysis. In this case, the results are promising regarding the detection of damage, as reported in Figure 25.

However, it should be pointed out that the percent reduction in modulus of elasticity to simulate damage is applied to a single, rather narrow, and localized slice of vertical elements. Thus, the damage is rather sharp on the model, while in reality, the damage, e.g., by degradation of the mechanical properties of an element, is often more widespread. In addition, the ability of the method to localize the damage on the bridge when close to the measurement point suggests the application of a diffuse monitoring system, such as distributed fiber-optic sensors.

## 4. Conclusions

This paper is focused on a methodology for damage detection and localization in bridges under both traffic loading and environmental variability by modeling different damage and temperature scenarios. The presented methodology is based on time-series data collected from a finite-element model.

Due to the non-stationary nature of recorded data, the fast Fourier transform is not applicable. Instead, the vertical acceleration measurements obtained from six sensors spaced along the bridge-like structure were analyzed using the Hilbert–Huang transform with variational model decomposition.

To remove the temperature effects from the damage-sensitive features, principal component analysis with dimensionality reduction was applied. It was observed that the first principal component with the highest variance in the data matrix was responsible for the temperature-related effects. As a result, the damage-sensitive features were reconstructed by removing the first principal component.

Finally, to detect and localize the damage, a machine learning algorithm (K-means) was applied. Using the symbolic data to reduce the amount of data, a technique of moving the time window was used for damage-sensitive features. A confidence boundary was deployed to evaluate damage indices values for each window, and a detection index was then defined to consolidate a result with high confidence.

Results showed that K-means in combination with principal component analysis with dimensionality reduction can identify and localize damages even with changing temperatures and analyze non-stationary vibrations due to traffic in the bridge. The methodology showed its effectiveness and reliability in the field of bridge structural health monitoring and damage detection and localization, under both traffic-induced loads and temperature changes.

This paper is the first step in this direction, but consequently, several limitations are still present, such as the use of a numerical example and not a real bridge due to the lack of vibration data under health and damage conditions and with varying temperatures, the simple modeling of the vehicle as a moving load, and the crossing of only one vehicle present in the bridge. Therefore, further steps of the research will consider different damage levels and locations, multiple damage states, and a real-world case study of a bridge structure to test the proposed methodology in different conditions under operational and environmental effects.

## Figures and Tables

**Table 1:** Geometry and mechanical properties of the numerical bridge superstructure.

Geometry (Units)	Symbol	Value
Left-span length (m)	*L*_1_	10
Right-span length (m)	*L*_2_	10
Total bridge length (m)	*L*	20
Bridge height (m)	*h*	0.6
Bridge width (m)	*t*	10
Material properties at T=20 °C		
Mass density (kg/m^3^)	*ρ*	2400
Young’s modulus (GPa)	E	35
Poisson’s ratio	*ν*	0.2
Shear modulus (GPa)	G	14.6

**Table 2:** Horizontal stiffness, *k_x_*, and vertical stiffness, *k_y_*, adopted for the three spring supports.

Stiffness (Units)	Left Support	Mid Support	Right Support
*k_x_* (N/m)	10,714,200	19,285,500	10,714,200
*k_y_* (N/m)	10^15^	10^20^	10^15^

**Table 3:** Sensor locations along the bridge.

Sensor	Characteristics	Position on the Beam Neutral Axis (y = 0.3 m)
S-01	¼ L_1_ from left-hand support	x = 2.5 m
S-02	½ L_1_ from left-hand support	x = 5.0 m
S-03	¾ L_1_ from left-hand support	x = 7.5 m
S-04	¾ L_2_ from right-hand support	x = 12.5 m
S-05	½ L_2_ from right-hand support	x = 15.0 m
S-06	¼ L_2_ from right-hand support	x = 17.5 m

**Table 4:** Damage scenarios provided by the benchmark.

Damage Scenarios	Damaged Elements	Location of Damage
Undamaged Scenario (UND)	0	
Damage Scenario 1 (DMG1)	2	At 1/2 L_1_ from left-hand support, starting from the bottommost edge
Damage Scenario 2 (DMG2)	4	
Damage Scenario 3 (DMG3)	6	
Damage Scenario 4 (DMG4)	2	At L_1_ from left-hand support, starting from the uppermost edge
Damage Scenario 5 (DMG5)	4	
Damage Scenario 6 (DMG6)	6	

**Table 5:** VMD parameters for each sensor.

Parameters	S01	S02	S03	S04	S05	S06
Number of modes, *K*	6	5	6	6	5	6
$\mathrm{Relative}\;\mathrm{tolerance},\;\varepsilon_{r}$	$10^{-5}$	$10^{-5}$	$10^{-5}$	$10^{-5}$	$10^{-5}$	$10^{-5}$
Penalty factor, α	1000	1000	1000	1000	1000	1000
Fidelity coefficient, 𝜏	0.1	0.1	0.1	0.1	0.1	0.1
$\mathrm{Absolute}\;\mathrm{tolerance},\;\varepsilon_{a}$	0.1	0.1	0.1	0.1	0.1	0.1
Number of iterations, O	100,000	100,000	100,000	100,000	100,000	100,000

**Table 6:** Method for choosing initial cluster centroid positions.

“cluster”	Perform a preliminary clustering phase on a random 10% subsample of X when the number of observations in the subsample is greater than k. This preliminary phase is itself initialized using “sample”. If the number of observations in the random 10% subsample is less than k, then the software selects k observations from X at random.
“plus” (default)	Select *k* seeds by implementing the *k*-means++ algorithm for cluster center initialization.
“sample”	Select k observations from X at random.
“uniform”	Select k points uniformly at random from the range of X. Not valid with the Hamming distance.
“numeric matrix”	k-by-*p* matrix of centroid starting locations. The rows of *Start* correspond to seeds. The software infers k from the first dimension of *Start*, so you can pass in [] for *k*.
“numeric array”	*k*-by-*p*-by-*r* array of centroid starting locations. The rows of each page correspond to seeds. The third dimension invokes replication of the clustering routine. Page *j* contains the set of seeds for replicate *j*. The software infers the number of replicates (specified by the “Replicates” name-value pair argument) from the size of the third dimension.
